# Sexual conflict over sex—an underappreciated consequence of childbirth?

**DOI:** 10.1093/emph/eoae027

**Published:** 2024-10-21

**Authors:** C Ruth Archer, Meaghan Castledine, David J Hosken

**Affiliations:** Institute of Evolutionary Ecology and Conservation Genomics, University of Ulm, Albert-Einstein-Allee 11, 89081 Ulm, Germany; Science and Engineering Research Support Facility (SERSF), University of Exeter, Penryn Campus, Penryn, Cornwall, TR10 9FE, UK; Science and Engineering Research Support Facility (SERSF), University of Exeter, Penryn Campus, Penryn, Cornwall, TR10 9FE, UK

**Keywords:** dyspareunia, inter-birth intervals, postpartum, sexual conflict, sexual dysfunction

## Abstract

Many postpartum women experience sexual dysfunction, characterised by reduced sexual motivation and libido, and pain during intercourse. Menstruation is also suppressed in breastfeeding women (lactational amenorrhoea). Lactational amenorrhoea has been discussed in an evolutionary context due to its positive impacts on birth spacing. In contrast, postpartum sexual dysfunction has not been viewed through an evolutionary lens. Might postpartum sexual dysfunction also be under selection? We discuss possible evolutionary explanations for postpartum sexual dysfunction. In particular, we suggest that sexual conflict, a widespread phenomenon that occurs when the evolutionary interests of males and females diverge, may be a cause of disrupted postpartum sex. This sexual conflict-based explanation generates predictions relevant to the health and well-being of new mothers that warrant testing.

## INTRODUCTION

While there is tremendous variation in how giving birth affects women’s sexual function, sexual activity is often disrupted postpartum. Postpartum women frequently have lower libido and arousal, struggle to orgasm, and experience painful intercourse (dyspareunia). These changes (hereon, postpartum sexual dysfunction) are common: one study reported that 85.7% of sexually active women surveyed at 18 months postpartum experienced dyspareunia when first resuming vaginal sex after giving birth, with approximately one in five women still experiencing this pain [1]. Ten per cent of these women described dyspareunia as ‘distressing’, ‘horrible’, or ‘excruciating’ [1].

Sexual dysfunction postpartum can have many causes including depressive symptoms, low partnership quality, stress, exhaustion, feeling unattractive, negative childbirth experiences, as well as perineal damage incurred during labour [2]. One key driver is breastfeeding and the hormonal changes associated with it [2]. In a meta-analysis collating data from 11 354 women, 48% of those breastfeeding experienced dyspareunia compared to 33% who were not breastfeeding [3]. In a smaller survey (*N* = 330), antenatal partnership quality and breastfeeding explained 24.3% of variance in female sexual function at 4 months postpartum [4].

Breastfeeding also suppresses ovulation (lactational anovulation) and menstruation (lactational amenorrhoea) [5]. This suppression means that breastfeeding women are less likely to conceive, and so extends inter-birth intervals [6]. This is important because short inter-birth intervals have costly effects on maternal and child health [7]; across large parts of the world, inter-birth intervals of less than 36 months are associated with higher infant mortality [8]. Accordingly, there appears to be a consensus that lactational anovulation/amenorrhoea are adaptations that extend inter-birth intervals in primates with slow rates of reproduction [9]. Sexual dysfunction has, however, to the best of our knowledge, not been discussed in light of evolutionary theory. If postpartum sexual dysfunction increases inter-birth intervals, might it also be evolutionarily advantageous?

## SEXUAL DYSFUNCTION THROUGH AN EVOLUTIONARY LENS

From an evolutionary perspective, the null hypothesis is that postpartum sexual dysfunction is a byproduct of other physiological and environmental changes associated with childbirth and is not under selection. Alternatively, postpartum sexual dysfunction might not be under direct selection, but positively correlated with a trait that is (e.g. lactational amenorrhoea). In this case, postpartum sexual dysfunction and lactational amenorrhoea would be correlated and the latter (but not the former) would improve maternal fitness by extending inter-birth intervals. In both scenarios, postpartum sexual dysfunction is selectively neutral.

If lactational anovulation/amenorrhoea alone are efficient barriers to conception (i.e. without postpartum sexual dysfunction), then sexual function changes could be maladaptive. This could happen because sexual and relationship satisfaction are, to some degree, linked [10]. Postpartum sexual dysfunction might therefore decrease relationship satisfaction and in turn, promote pair separation. How separation affects maternal fitness depends on offspring survival within/outside of pair bonds and how likely mothers are to recouple. A meta-analysis suggested that paternal death affected child survival in ~32% of studies [11] and re-partnering following separation or widowhood is common in both sexes [12]. Moreover, lactational anovulation/amenorrhoea are not absolute barriers to contraception—women can ovulate when exclusively breastfeeding [5]. For these reasons, this explanation seems tenuous.

An adaptive explanation for disrupted sex postpartum is that the costs of short inter-birth intervals are high for both sexes and selection has favoured multiple barriers to conception postpartum (e.g. sexual dysfunction and lactational amenorrhoea) because no single barrier is perfect and each additional barrier decreases the likelihood of conception. This predicts that inter-birth intervals are longer when both processes operate rather than either alone and both sexes improve their fitness as a result. This scenario relies on males and females ‘agreeing’ (in an evolutionary sense) on optimal inter-birth intervals. If not, there would be sexual conflict over intervals and therefore over mating and postpartum sex. Sexual conflict would also provide an explanation for the redundancy in the means of extending inter-birth intervals (i.e. postpartum sexual dysfunction reduces the likelihood that postpartum women want to have sex, while lactational anovulation and amenorrhoea reduce fertilization success should sex occur).

Sexual conflict is rife in nature [13–15]. It occurs when the evolutionary interests of males and females do not align, either because the sexes (Box 1) differ in their optimal outcomes of an interaction (interlocus sexual conflict) or in their optimal values of a shared trait (intralocus sexual conflict) [13–15]. Hip width is a regularly cited example of the latter—alleles for wider hips have a selective advantage in women (due to childbirth), but may be costly in men (reduced mobility), meaning that genes influencing hip widths are under contrasting patterns of selection when expressed in either sex [15]. Interlocus sexual conflict occurs over the outcomes of intersexual interactions. For example, selection favours males who can bias interactions in their favour and in turn, females who evolve counter-adaptations to male manipulation (or *vice versa*). Cycles of sexually antagonistic trait evolution can ensue [16].

BOX 1: **TERMINOLOGY AND DEFINITIONS**

In discussing the evolution of postpartum sexual dysfunction, we need a definition of sex that is applicable to all species. Accordingly, we use the biological definition, based on the one universal difference between females and males—gamete size [43]. Females produce larger gametes (eggs) and males produce smaller gametes (sperm). Because we use this definition, we discuss cis-gender, heterosexual couples. This does not capture the full diversity of human sexuality and gender but allows us to draw cross-species comparisons.

Mating frequency is a major focus of interlocus sexual conflict in nature [13]. This is because females tend to maximize their fitness by investing more in each reproductive event, while males maximize their fitness by engaging in more reproductive events and investing relatively little in each. Additional copulations therefore generally increase male fitness more than they increase female fitness, and so optimal mating rates tend to be higher in males [13]. Thus, one explanation for postpartum women exhibiting multiple barriers to conception is that there is (or has been in human evolutionary history) sexual conflict over the recommencement of mating and ultimately inter-birth intervals. If females can be coerced into copulating earlier than the female fitness optimum, pre-copulatory barriers to fertilization (e.g. reduced libido) would not be sufficient to thwart male’s evolutionary interests and post-copulatory barriers (e.g. suppressed ovulation) would evolve. Note that these are evolutionary arguments and do not require conscious planning or moral foundations. If postpartum sexual dysfunction results from sexual conflict, we would expect to see sex-specific optimal patterns of postpartum sexual activity and associated traits such as inter-birth intervals or family size. Primates offer insight into the potential for sexual conflict over these characters.

## SEXUAL CONFLICT OVER MATING IN PRIMATES

Many non-human primates experience lactational amenorrhoea, including great apes such as gorillas and chimpanzees, and Old World monkeys such as baboons and rhesus monkeys [17]. In these Old World primates, evidence for sexual conflict over sexual activity is striking [18]. Following male takeovers (when males displace rivals as female suitors) or shifts in dominance hierarchies, males in many species frequently kill offspring that are unrelated to them [19]. This is because short inter-birth intervals may mean that females have to care for two offspring synchronously. The high costs of this select for lactational amenorrhoea, and in turn, for male traits to bypass this barrier to conception (i.e. infanticide), and bring females into oestrus sooner. Thus, infanticide is an extreme and widespread manifestation of sexual conflict over mating [9].

Sexually selected foeticide, where male harassment of females induces pregnancy loss and returns females to a fertile state sooner, is another example of primate sexual conflict over reproductive timing. Foeticide is hard to observe, but theory suggests that in many primates species, males are under stronger selection to commit foeticide than infanticide [20]. Similarly, in some species, females terminate pregnancies after olfactory, visual, auditory, or tactile exposure to non-sire males (the Bruce effect). The Bruce effect may reduce the costs paid by females who are likely to experience foeticide or infanticide should their pregnancy progress [20]. In which case, this process is also driven by sexual conflict over reproductive scheduling. Data are needed to understand the prevalence of these diverse forms of conflict and to relate their incidence to primate ecology and mating systems.

While male takeovers are associated with sexual conflict over mating and inter-birth intervals in many primates, monogamy may have evolved to mitigate this conflict by better aligning the evolutionary interests of males and females [21]. Given that social monogamy is widespread in contemporary human populations, has the potential for sexual conflict over inter-birth intervals and postpartum sexual activity vanished?

## IS THERE THEORETICAL SUPPORT FOR SEXUAL CONFLICT OVER POSTPARTUM REPRODUCTION IN HUMANS?

If (genetic) monogamy is perfect, life-long and no other mating opportunities exist outside of bonded pairs, the evolutionary interests of the sexes will align and sexual conflict over mating would be absent. However, human mating systems are diverse; monogamous, polyandrous, polygynous and shifting mating patterns are all evident across cultural groups [22]. While long-term pair bonds may have been the dominant mating system in human history, these bonds can change over time – that is, there is serial monogamy. Moreover, social monogamy does not always equate to sexual monogamy; in the traditional semi-nomadic pastoral Himba population for example, 31.8% of women had one or more extra-pair births during their lifetimes [23]. Moreover, human testis size suggests an evolutionary history that is not strictly monogamous [24]. Accordingly, the potential for sexual conflict in human mating systems remains.

Sexual conflict over postpartum sexual activity is most likely if a woman’s current partner did not father her existing children. This is because incoming males do not experience reductions in their own fitness via deleterious effects of short inter-birth intervals on existing children. Accordingly, if a man’s relatedness to a partner’s current offspring is less than his relatedness to any future offspring, we expect men to prefer (in an evolutionary sense) shorter inter-birth intervals than when the reverse is true [25]. However, the effects of female mate switching on optimal male inter-birth intervals are complex. If paternity certainty declines rapidly after the birth of the first child, a male may favour longer inter-birth intervals than his mate, to buffer his offspring from competition for maternal investment with future unrelated siblings [26]. If paternity certainty declines at an intermediate tempo after childbirth, men may favour shorter inter-birth intervals than women to take advantage of another reproductive opportunity with their current mate [26]. Both of these male strategies have fitness costs for females. That is, there is sexual conflict over inter-birth intervals when women re-partner on a short/intermediate timescale [26].

Within pair-bonded couples, sexual conflict over postpartum copulation seems most likely if there is sexual conflict over optimal family size, and if men tend to (or have historically tended to) prefer larger families than women. This preference difference seems feasible because the costs of child production are greater for women than men. However, models assuming serial monogamy have shown that shorter inter-birth intervals (correlated with larger family size) will not consistently improve male fitness [26]. Instead, only men who are preferred as mates and can readily re-partner if their current mate dies during childbirth are likely to benefit from shorter inter-birth intervals than women [26]. However, even if only ‘high-quality’ men benefit by having significantly larger families, these men would disproportionately contribute to the gene pool and the ‘trait’ (preference for larger families/short inter-birth intervals) would spread. Whether this happens depends on how heavily reproductive success is skewed towards these males.

Whether theory predicts sexual conflict over inter-birth intervals, which might equate to sexual conflict over the resumption of postpartum sex, is complicated. It depends on factors including paternity assurance, the frequency and tempo at which pair bonds are dissolved and new bonds re-established, and by whom. In contemporary populations, these traits vary. Moreover, a lack of contemporary sexual conflict over family size/inter-birth intervals does not mean the absence of sexual conflict over these traits in our evolutionary history. Many factors that influence the potential for conflict have shifted in our recent evolutionary past; could a historic tendency for larger families [27] or higher rates of male mortality (see [28]) have exacerbated conflict? Modelling this complexity is challenging. Data from contemporary populations however, show signs of sexual conflict over postpartum sex.

## ARE DATA CONSISTENT WITH SEXUAL CONFLICT OVER POSTPARTUM SEX?

As highlighted above, sexual conflict over postpartum sex is most likely if men want larger families than women. While men often want more children than women, the magnitude of this difference varies between societies and in some cases, women want more children than men [26]. Thus, evidence for conflict over family size is equivocal.

The observation that male libido is often higher than female libido postpartum [29] and female, but not male, libido typically declines postpartum [30] is more consistent with sexual conflict over postpartum sexual activity. However, this male-female discrepancy in libido is not universal [31] and many women report high libido postpartum [32]. Additionally, testosterone levels decline in new fathers, reflecting a hormonally mediated shift away from investment in mating and towards greater investment in parental care [33]. While these data suggest a general tendency for greater male sexual motivation postpartum compared to females, this alone is not a clear signal of sexual conflict over postpartum sex.

New mothers’ perceptions of their partner’s desire can predict the resumption of many sexual activities postpartum [34], and some women report resuming sexual activity postpartum expressly for the benefit of their partners [32, 35]. Women also report feeling pressured to have sex postpartum [35, 36], however, this may reflect internal/wider societal expectations [36]. Once more these data are consistent with sexual conflict over postpartum sexual activity but do not provide unequivocal evidence for it.

Strikingly, sexual violence appears elevated in the postpartum period. Macy *et al*. [37] tracked rates of psychological abuse, physical, and sexual violence across pregnancy and the postpartum period. The risk of psychological and sexual violence was greatest in the month following infant delivery [37]. Furthermore, other work has revealed that while sexual violence was the least common form of partner violence reported during and in the year following pregnancy, all incidences of sexual violence reported occurred in the first 40 days postpartum [38]. It was suggested that this apparent increase in sexual violence postpartum may be associated with reduced libido in new mothers [37]. This observation suggests sexual conflict over postpartum sex and that some males are attempting to control when sex resumes. This clearly has important ramifications for the well-being of new mothers.

The discussion above largely focuses on data from within pair-bonded couples. However, it is important to note that the potential for sexual conflict over inter-birth intervals may be greater when women switch partners between reproductive events [26]. This is supported by data; as in some Western populations stepfathers are more likely to perpetrate homicide against young children than biological fathers [39].

## CONCLUSION

Of all the possible explanations for why women experience postpartum sexual dysfunction, we focus on a sexual conflict-based explanation because this predicts (and therefore helps explain why) some new mothers are under pressure to resume postpartum sex and may be more vulnerable to sexual violence. For this reason, it is worth studying sexual conflict over postpartum sexual activity, and understanding what this means for the well-being and safeguarding of new mothers. This is not the only arena where sexual conflict theory provides important insights into human health [40], but given the incidence and impact of disrupted sexual function postpartum, the potential benefits to understanding this phenomenon through an evolutionary lens are significant.

More generally, discussing postpartum sexual dysfunction from an evolutionary perspective might help new parents. Selection has favoured a suite of mechanisms to extend intervals between births such as lactational anovulation, amenorrhoea, and (possibly) sexual dysfunction. Might communicating the possible functional significance of sexual dysfunction (i) promote help-seeking behaviours (especially given positive associations between health literacy and professional help-seeking for other conditions [41]), (ii) ease feelings of ‘guilt’ and ‘failure’ reported by new mothers due to libido declines postpartum [35], and (iii) adjust new parents’ (or wider societal) expectations about postpartum sex? The latter is important because women with partners who are understanding about reduced female libido postpartum have higher relationship satisfaction [42]. If so, better understanding and communicating why sex changes postpartum could promote maternal well-being and relationship satisfaction and ease some of the pressure on new mothers, regardless of the origins of that pressure.
